# Semi-metallic Be_5_C_2_ monolayer global minimum with quasi-planar pentacoordinate carbons and negative Poisson's ratio

**DOI:** 10.1038/ncomms11488

**Published:** 2016-05-03

**Authors:** Yu Wang, Feng Li, Yafei Li, Zhongfang Chen

**Affiliations:** 1 College of Chemistry and Materials Science, Jiangsu Key Laboratory of Biofunctional Materials, Nanjing Normal University, Nanjing, Jiangsu 210023, China; 2 Department of Chemistry, Institute for Functional Nanomaterials, University of Puerto Rico, Rio Piedras Campus, San Juan, Puerto Rico 00931, USA

## Abstract

Designing new materials with novel topological properties and reduced dimensionality is always desirable for material innovation. Here we report the design of a two-dimensional material, namely Be_5_C_2_ monolayer on the basis of density functional theory computations. In Be_5_C_2_ monolayer, each carbon atom binds with five beryllium atoms in almost the same plane, forming a quasi-planar pentacoordinate carbon moiety. Be_5_C_2_ monolayer appears to have good stability as revealed by its moderate cohesive energy, positive phonon modes and high melting point. It is the lowest-energy structure with the Be_5_C_2_ stoichiometry in two-dimensional space and therefore holds some promise to be realized experimentally. Be_5_C_2_ monolayer is a gapless semiconductor with a Dirac-like point in the band structure and also has an unusual negative Poisson's ratio. If synthesized, Be_5_C_2_ monolayer may find applications in electronics and mechanics.

## Abstract (web summary)

There is tremendous research into new materials with interesting topological properties. Here, the authors use density functional theory to design a two dimensional material pseudo-planar material in which each carbon atom is bonded to five beryllium atoms forming a quasi-planar pentacoordinate carbon moiety.

## Graphical abstract

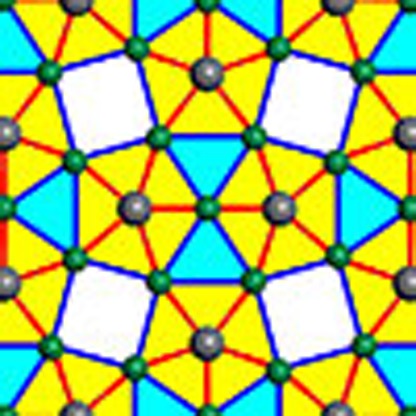

Carbon in known molecules and materials typically has tetrahedral tetracoordination (for example, diamond), planar tricoordination (for example, graphite) or linear dicoordination (for example, ethyne) arrangements. Especially, the tetrahedral preference of tetracoordinate carbon compounds, which was deduced by van't Hoff^1^ and Lebel^2^ more than a century ago, is one of the major foundations of organic chemistry. In 1968, Monkhorst^3^ first discussed an exceptional bonding pattern of carbon, namely planar tetracoordinate carbon (ptC), by proposing a planar methane (*D*_4h_), which is actually not a minimum energy structure. Later in 1970, by insightful analysis of ptC bonding in the hypothetical planar methane, Hoffman *et al*.^4^ suggested that ptCs can be stabilized electronically by replacing H atoms in planar methane by *σ* donors (to facilitate electron transfer to electron-deficient *σ* bonds) or *π* acceptors (to delocalize the unfavourable lone pair of ptC). Along this line, in 1976 Collins *et al*.^5^ theoretically devised the first ptC-containing molecule 1, 1-dilithiocyclopropane. One year later Cotton and Millar^6^ synthesized the first ptC-containing molecule, although the unique ptC configuration was not recognized at that time. Ever since, ptC chemistry has been a subject of extensive studies^7^,^8^,^9^. Numerous ptC species have been designed theoretically^10^,^11^,^12^,^13^,^14^,^15^ and some global minimum structures, such as CAl_4_^+^ (ref. 16), CAl_4_^2+^ (ref. 17) and CAl_3_Si^−^ (ref. 18) have been observed experimentally. More excitingly, besides ptC, many molecules containing planar pentacoordinate carbon (ppC)^19^,^20^,^21^,^22^ and hexacoordinate carbon (phC)^23^,^24^,^25^ have been designed computationally. The rule-breaking chemical bonding in these planar hypercoordinate carbons can lead to completely new molecules and materials, which are of fundamental importance to chemistry and materials science.

The unique topology and dimensionality may lead to exceptional properties of materials. It is not a surprise to witness the growing interest in designing ptC-containing solids and nanostructures in recent years^26^,^27^,^28^,^29^,^30^. Especially, stimulated by the extensive studies of graphene^31^,^32^ and inorganic layered materials^33^,^34^, many two-dimensional (2D) materials with rule-breaking chemical bonding have been designed computationally^35^,^36^,^37^,^38^,^39^,^40^,^41^,^42^. For example, on the basis of ptC molecule CB_4_ (ref. 43), Wu *et al*.^35^ designed the first ptC-containing 2D material, namely B_2_C graphene, which was later confirmed to be only a local minimum^44^. Li *et al*.^36^ and Dai *et al*.^37^ predicted that Al_2_C monolayer in its lowest-energy configuration have all C atoms being ideal ptC. More interestingly, Li *et al*.^45^ demonstrated recently that one C atom can bind to six beryllium (Be) atoms in an almost planar manner, yielding a phC-featuring 2D material with a Be_2_C stoichiometry. With intriguing structural and electronic properties, these 2D materials are expected to have important applications in some specific fields.

In contrast to ptC and phC, no attempt exists for extending ppC into solids until now. Motivated by the successful design of ptC- and phC-containing materials, we are quite curious whether it is possible to extend ppC into solids. Addressing this issue would deliver not only unique structures but also some fantastic properties, which is of both theoretical and practical importance.

In this work, by means of density functional theory (DFT) computations, we first confirm that the ppC molecule Be_9_C_2_^4−^ is a local minimum on the potential energy surface. Based on the structural characters of Be_9_C_2_^4−^, we design a ppC-containing 2D material, namely Be_5_C_2_ monolayer, in which each C atom binds to five Be atoms to form a quasi-planar pentacoordinate moiety. Our Be_5_C_2_ monolayer has rather good thermal and kinetic stabilities and is energetically the most favourable isomer in 2D space. Dramatically, the electronic band structure of Be_5_C_2_ monolayer has a Dirac-like point at the Fermi level, endowing an intriguing semimetallic feature. More interestingly, Be_5_C_2_ monolayer possesses a negative Poisson's ratio, which is rather rare in nanostructures. These fascinating properties make Be_5_C_2_ monolayer a promising candidate for future applications in electronics and mechanics.

## Results

### Be_9_C_2_^4−^ as the inspiring ppC species for 2D monolayer

**Figure 1:**
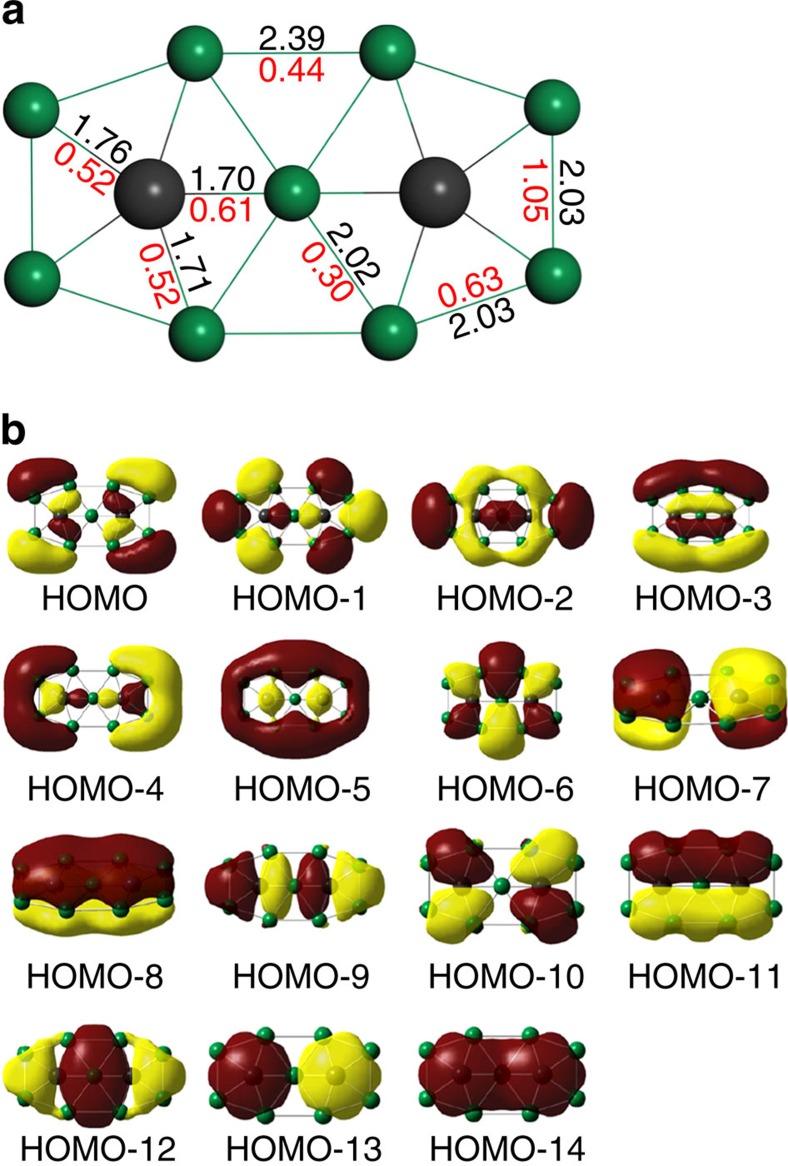
Be_9_C_2_^4−^ molecule. (**a**) Optimized geometric structure of Be_9_C_2_^4−^ molecule. The black and green balls represent C and Be atoms, respectively. The red and black numbers are the Wiberg bond index (WBI) and lengths (in angstroms) of the representative chemical bonds, respectively. (**b**) The canonical molecular orbitals of Be_9_C_2_^4−^.

Our design of periodic 2D Be_5_C_2_ monolayer is initially inspired by our finding of the ppC molecule, namely Be_9_C_2_^4−^, which has singlet ground state and *D*_2h_ symmetry (Fig. 1a). Computed at B3LYP level of theory with 6–311+G*(*d*, *p*) basis set, Be_9_C_2_^4−^ is a local minimum with the lowest vibrational frequency of 158.5 cm^−1^. In this molecule, each C atom binds to five Be atoms to form a ppC moiety of Be_5_C, which has been shown to be a local minima^20^. Structurally, Be_9_C_2_^4−^ can be viewed as two Be_5_C moieties fused by sharing one Be atom. The C–Be bond lengths are in the range of 1.70–1.76 Å, whereas the Be–Be bond lengths are in the range of 2.02–2.39 Å.

According to the natural population analysis, each ppC of Be_9_C_2_^4−^ possesses a 2.18 |e| negative charge (−0.40 |e| according to the Hirshfeld charge population analysis) and the natural electron configuration is 2*s*^1.42^2*p*_*x*_^1.47^2*p*_*y*_^1.66^2*p*_*z*_^1.60^, indicating that ppCs in Be_9_C_2_^4−^ are essentially stabilized through Be *σ*-donation and delocalization of carbon 2*p*_*z*_ electrons. The computed Wiberg bond index for C–Be bonds are 0.52 and 0.61, respectively, resulting in a total Wiberg bond index of 2.81 for each ppC. Moreover, we also scrutinized the molecular orbitals of Be_9_C_2_^4−^ to get more information on its stabilization mechanism. As shown in Fig. 1b, the highly delocalized *π* (for example, HOMO-7 and HOMO-8) and *σ* (for example, HOMO-3, HOMO-4 and HOMO-5) orbitals can help maintain the planar configuration.

### Geometric properties of Be_5_C_2_ monolayer

**Figure 2:**
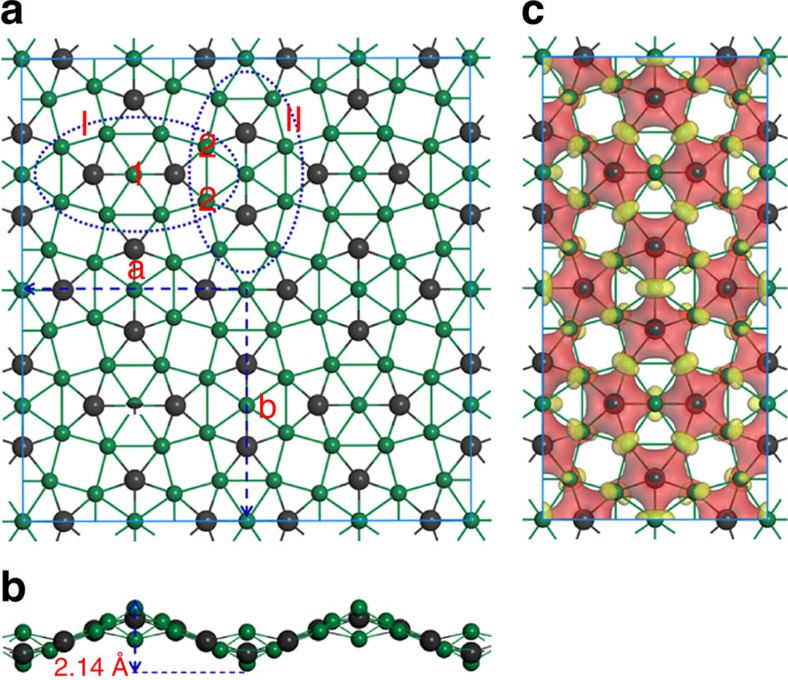
Geometric and electronic structures of Be_5_C_2_ monolayer. (**a**) Top and (**b**) side views of geometric structure of Be_5_C_2_ monolayer. The blue dashed lines denote a unit cell; a and b represent the lattice vectors, 1 and 2 denote the different Be atoms, I and II represent the different Be_9_C_2_ moieties. (**c**) Deformation charge density of 2D Be_5_C_2_ monolayer. Red and yellow refer to electron accumulation and depletion regions, respectively.

The fusing of two Be_5_C moieties to Be_9_C_2_^4−^ reminds us of the roadmap of fusing benzene rings to polycyclic aromatic hydrocarbons (for example, naphthalene and anthracene) and then to 2D infinite graphene. Inspired by the ppC-containing Be_9_C_2_^4−^, we designed a new 2D inorganic material, namely Be_5_C_2_ monolayer by generalizing the structural characters of Be_9_C_2_^4−^. As shown in Fig. 2a, 1 unit cell of Be_5_C_2_ monolayer consists of 8 C atoms and 20 Be atoms with the optimized lattice parameters being *a*=8.92 Å and *b*=9.21 Å, respectively. Similar to Be_9_C_2_^4−^, in Be_5_C_2_ monolayer each C atom binds to five Be atoms to form a ppC moiety of Be_5_C and two neighbouring Be_5_C moieties share one Be atom (Be_1_) to form a Be_9_C_2_ moiety in the *a* (Be_9_C_2_-I) or *b* (Be_9_C_2_-II) direction. Especially, one Be_9_C_2_-I moiety is fused with four neighbouring Be_9_C_2_-II moieties by sharing the peripheral Be atoms (Be_2_) and *vice versa*, leading to the formation of a 2D network with four Be_9_C_2_ moieties in one unit cell. The optimized coordinates of Be_5_C_2_ monolayer are presented in Supplementary Table 1. It is noteworthy that the Be_5_C_2_ monolayer is remarkably buckled rather than an exactly planar structure (Fig. 2b). The buckling, measured by the vertical distance between the bottommost Be atoms and the uppermost Be atom, is as high as 2.14 Å. Even so, the ppCs in Be_5_C_2_ monolayer still have a good planarity (Supplementary Fig. 1). According to our computations, the total degrees of five Be–C–Be angles for ppCs in Be_9_C_2_-I and Be_9_C_2_-II moieties are 371.44° and 364.84°, respectively, which are a little higher than the ideal 360°. Interestingly, when Be_9_C_2_^4−^ is neutralized by four protons (H^+^), the obtained Be_9_C_2_H_4_ molecule is also severely buckled (Supplementary Fig. 2). In Be_5_C_2_ monolayer, the length of C–Be_1_ bonds (1.66 Å, 1.68 Å) is much shorter than that of C–Be_2_ bonds (1.73 Å, 1.74 Å). Moreover, our computations revealed that Be_5_C_2_ monolayer has a non-magnetic ground state, indicating that there are no unpaired electrons in Be_5_C_2_ monolayer.

We then computed the deformation electronic density of Be_5_C_2_ monolayer to elucidate its bonding nature. The deformation electronic density is defined as the total electronic density excluding those of isolated atoms. As clearly shown in Fig. 2c, some electrons are extracted from the 2*s* orbitals of Be atoms and well delocalized over C–Be bonds, indicating that C atoms form multicentre covalent bonds with neighbouring Be atoms, which is crucial for stabilizing the ppC moieties. The similar stabilizing mechanism has been found in ptC- and phC-containing 2D materials^35^,^36^,^37^,^38^,^39^,^40^,^45^. According to the Hirshfeld charge popular analysis, C, Be_1_ and Be_2_ atoms in Be_5_C_2_ monolayer possess a −0.32, +0.16 and +0.12 |e| charge, respectively. The buckling of Be_5_C_2_ monolayer stretches the Be–Be distances and probably helps reduce the otherwise even stronger repulsive interactions between Be atoms.

We also used the recently developed Solid State Adaptive Natural Density Partitioning (SSAdNDP) method^46^ to better understand the unique chemical bonding of Be_5_C_2_ monolayer. According to our results (Supplementary Fig. 3), there is no classical two-centre–two-electron (2c–2e) C–Be bond in Be_5_C_2_ monolayer. For one unit cell of Be_5_C_2_ monolayer, the SSAdNDP search revealed twenty-four 3c–2e Be–C–Be *σ*-bonds (responsible for the bonding within the Be_5_C units), four 4c–2e *σ*-bonds on four Be squares and eight 6c–2e *π*-bonds over eight Be_5_C units, accounting for 72 electrons per unit cell. This bonding pattern is consistent with the symmetry of Be_5_C_2_ monolayer. Especially, the existence of delocalized *σ*- and *π*-bonds could essentially stabilize the ppCs in Be_5_C_2_ monolayer.

### Stability of Be_5_C_2_ monolayer

Although Be_5_C_2_ monolayer has rather intriguing structural properties, we are unclear whether it is a stable structure. To assess the stability, we first computed the cohesive energy of Be_5_C_2_ monolayer, which is defined as: *E*_coh_=(*nE*_C_+*mE*_Be_−*E*)/(*n+m*), in which *E*_C_, *E*_Be_ and *E* are the total energies of a single C atom, a single Be atom and Be_2_C monolayer, respectively; *n* and *m* are the number of C and Be atoms in the supercell, respectively. According to our computations, Be_5_C_2_ monolayer has a cohesive energy of 4.58 eV per atom. As a reference, the cohesive energies of the experimentally realized 2D silicene^47^,^48^ and phosphorene^49^,^50^ are 3.71 and 3.61 eV per atom, respectively. As silicene and phosphorene are composed of covalent bonds, the even higher cohesive energy can ensure that Be_5_C_2_ monolayer is a strongly connected network.

**Figure 3:**
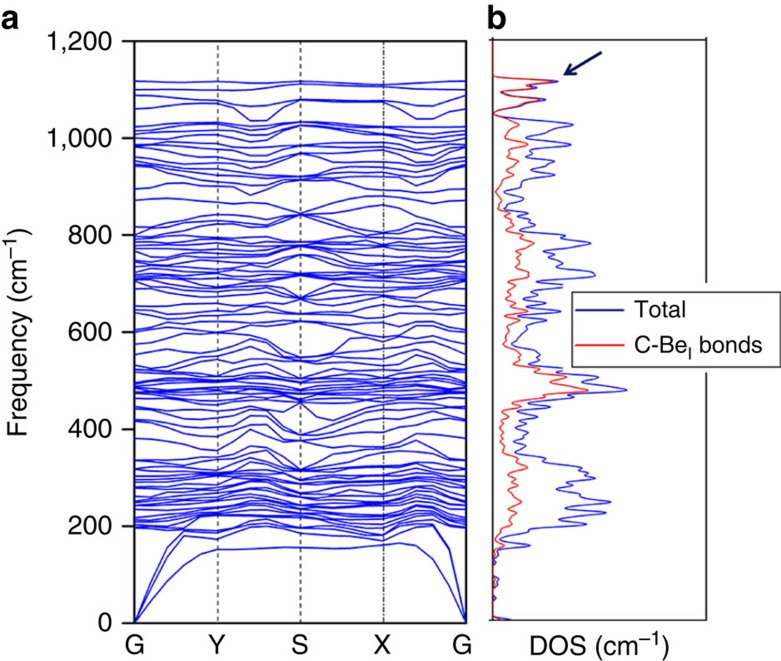
Kinetic stability of Be_5_C_2_ monolayer. (**a**) Phonon spectrum and (**b**) phonon DOS of Be_5_C_2_ monolayer.

The stability of Be_5_C_2_ monolayer can be further confirmed by its phonon dispersion curves. As shown in Fig. 3a, no appreciable imaginary phonon mode is present, suggesting the good kinetic stability of Be_5_C_2_ monolayer. Remarkably, the highest frequency of Be_5_C_2_ monolayer reaches up to 1,120 cm^−1^, which is higher than those of MoS_2_ monolayer (473 cm^−1^)^51^, silicene (580 cm^−1^)^52^ and our recently designed phC-containing Be_2_C monolayer (1,020 cm^−1^)^45^, indicating robust C–Be bonds in Be_5_C_2_ monolayer. The analysis of partial phonon density of states (DOS; Fig. 3b) revealed that the highest frequency of Be_5_C_2_ monolayer is mainly contributed by C–Be_1_ bonds.

Moreover, to examine the thermal stability of Be_5_C_2_ monolayer, we performed first-principles molecular dynamic (FPMD) simulations using a 2 × 2 supercell. Our three simulations at temperature of 1,000, 1,500 and 2,000 K show that Be_5_C_2_ monolayer can maintain its structural integrity throughout a 10-ps FPMD simulation up to 1,500 K, but is seriously disrupted at 2,000 K, suggesting that Be_5_C_2_ monolayer has a melting point between 1,500 and 2,000 K (Supplementary Fig. 4). We also performed optimizations for the distorted structures from MD simulations. After full atomic relaxation, those structures from MD simulations at 1,000 and 1,500 K can recover the initial configuration of Be_5_C_2_ monolayer. The above results demonstrate that Be_5_C_2_ monolayer has a remarkable thermal stability.

**Figure 4:**
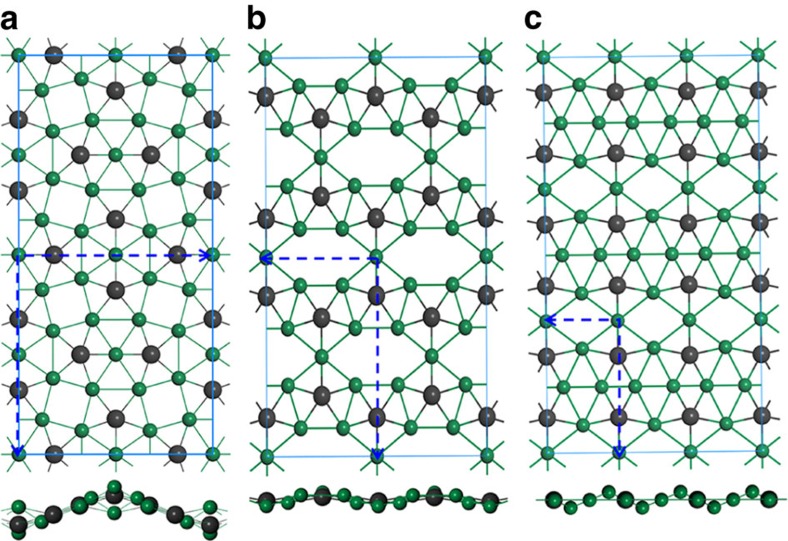
Low-energy isomers. Geometric structures of Be_5_C_2_-I (**a**), Be_5_C_2_-II (**b**) and Be_5_C_2_-III (**c**). The blue dashed lines denote unit cells.

The moderate cohesive energy, all positive phonon modes and good thermal stability can ensure that Be_5_C_2_ monolayer is at least a local minimum structure on the potential energy surface. However, is the ppC-containing Be_5_C_2_ monolayer a global minimum? It is noteworthy that the global minimum structure is more likely to be achieved than the local minimum structures experimentally. For example, many isomers of graphene, such as Haeckelite graphene^53^, T-graphene^54^ and penta-graphene^55^ have been designed computationally, but none of them has been realized experimentally. Therefore, we performed a global search for the lowest-energy structure of Be_5_C_2_ monolayer in the 2D space using first-principles-based particle-swarm optimization (PSO) method as implemented in CALYPSO code. After a comprehensive search, we obtained three stable isomers of 2D Be_5_C_2_, which are labelled as Be_2_C_5_-I, Be_2_C_5_-II and Be_2_C_5_-III, respectively. As shown in Fig. 4a, Be_2_C_5_-I is actually the above discussed ppC-featuring Be_5_C_2_ monolayer. Interestingly, in Be_2_C_5_-II (Fig. 4b) and Be_2_C_5_-III (Fig. 4c), each C atom binds to five Be atoms to form a ppC moiety of Be_5_C and the Be_9_C_2_ moieties also can be found in these two isomers. Be_2_C_5_-I is 50 and 101 meV per atom lower in energy than Be_2_C_5_-II and Be_2_C_5_-III, respectively, indicating that Be_2_C_5_-I is the global minimum structure in the 2D space. Therefore, the ppC-featuring Be_5_C_2_ monolayer holds great potential to be realized experimentally.

### Electronic properties of Be_5_C_2_ monolayer

**Figure 5:**
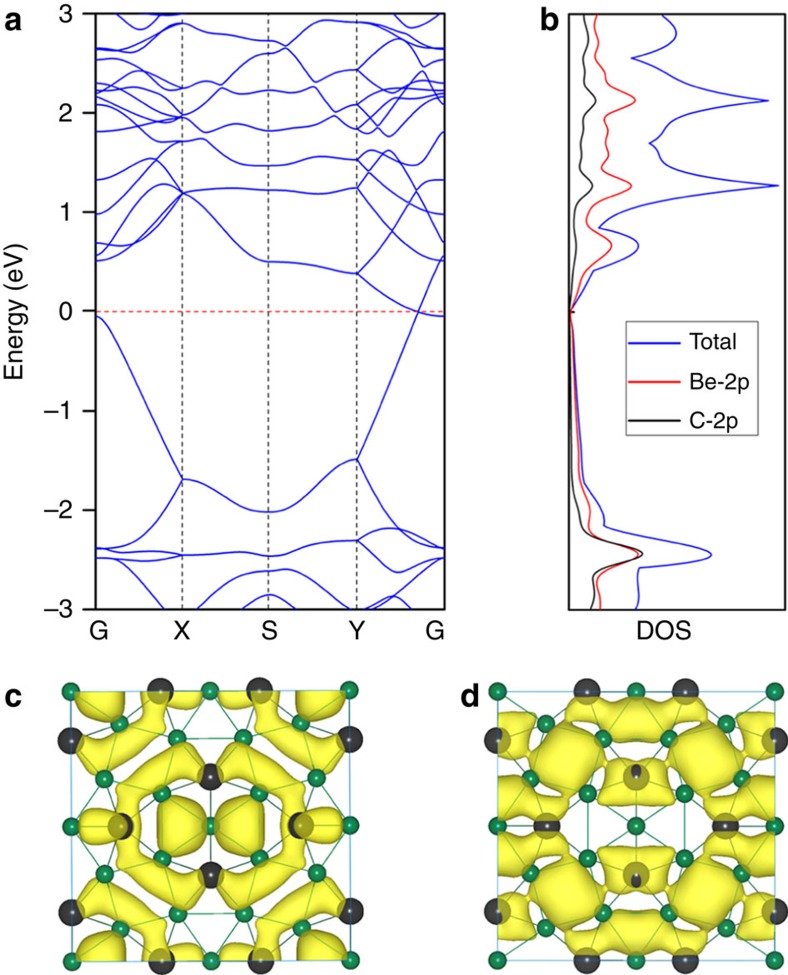
Electronic properties of Be_5_C_2_ monolayer. (**a**) Band structure and (**b**) DOS of Be_5_C_2_ monolayer. The Fermi level is assigned at 0 eV. (**c**,**d**) The isosurfaces of partial charge densities for the (**c**) VBM and (**d**) CBM of Be_5_C_2_ monolayer. The isovalue is 0.015 e A^−3^.

With such interesting structural characteristics, does Be_5_C_2_ monolayer also have intriguing properties? To address this issue, we computed the band structure of the lowest-energy Be_5_C_2_ monolayer. As shown in Fig. 5a, Be_5_C_2_ monolayer is gapless or semi-metallic with the conduction band minimum (CBM) and valence band maximum (VBM) meeting at the Fermi level, which is quite similar to that of graphene. However, the meeting point of Be_5_C_2_ monolayer is located on the path from G (0, 0, 0) point to Y (0, 0.5, 0) point rather than on a high-symmetry point as for graphene. Especially, the conduction and valence bands around the Fermi level exhibit a linear dispersion relation, suggesting that the meeting point of Be_5_C_2_ monolayer is also Dirac-like. Considering that the PBE functional tend to underestimate the band gap, we recomputed the band structure of Be_5_C_2_ monolayer using the hybrid HSE06 functional^56^ and found that the dispersion of the valence and conduction bands at the Fermi level is similar to that predicted by PBE and no appreciable band gap can be identified (Supplementary Fig. 5). Thus, the gapless property of Be_5_C_2_ monolayer is solid.

To obtain deeper insight into the electronic properties of Be_5_C_2_ monolayer, we analysed its DOS. As shown in Fig. 5b, the DOS is zero at the Fermi level, which further supports the presence of the Dirac cone. The partial DOS analysis shows that the VBM and CBM are contributed by both Be-2*p* and C-2*p* states, and the contribution from Be-2*p* states is much more than that from C-2*p* states. Moreover, we plotted the partial charge densities of the VBM and CBM. As shown in Fig. 5c,d, both VBM and CBM are mainly originated from the delocalized orbitals of Be atoms and partially from the multicentre bonding between C and Be atoms.

### Mechanical properties of Be_5_C_2_ monolayer

Besides the electronic properties, we also investigated the mechanical properties of Be_5_C_2_ monolayer by examining its elastic constants. As a validation, the computed elastic constants of graphene are C_11_=C_22_=342.93 GPa and C_12_=C_21_=62.23 GPa respectively, which achieve good agreements with experimental measurements^57^ and previous computations^58^. For Be_5_C_2_ monolayer, its elastic constants were computed to be C_11_=32.90 GPa, C_22_=130.89 GPa, C_12_=C_21_=−5.32 GPa and C_66_=48.32 GPa, which are in agreement with the mechanical stability criteria for a tetragonal 2D sheet (C_11_C_22_−C_12_^2^>0, C_66_>0)^55^. The in-plane Young's modules along *a* (Y_*a*_) and *b* (Y_*b*_) directions, which can be deduced from the elastic constants by Y_*a*_=(C_11_C_22_−C_12_C_21_)/C_22_ and Y_*b*_=(C_11_C_22_−C_12_C_21_)/C_11_, were computed to be 32.68 and 130.03 N m^−1^, respectively. As Y_*a*_ is not equal to Y_*b*_, Be_5_C_2_ monolayer is mechanically anisotropic. Moreover, computed at the same level of theory, the in-plane Young's modules of Be_5_C_2_ monolayer are less than those of graphene (Y_*a*_=Y_*b*_=331.63 N m^−1^) but higher than those of phosphorene (Y_*a*_=25.50 N m^−1^ and Y_*b*_=91.61 N m^−1^), suggesting that Be_5_C_2_ monolayer has good mechanical properties.

Remarkably, we noted that Be_5_C_2_ monolayer has a negative C_12_, which results in negative Poisson's ratios of −0.041 (C_12_/C_22_) and −0.16 (C_12_/C_11_) for *a* and *b* directions, respectively. It is noteworthy that the Poisson's ratio is defined as the negative ratio of transverse to axial strain. The negative Poisson's ratio indicates that Be_5_C_2_ monolayer can be compressed or stretched in both two directions at the same time. For a validation, we applied a uniaxial strain of 5% in *a* and *b* directions of Be_5_C_2_ monolayer, respectively. Just as expected, the equilibrium lattice parameters of *b* and *a* directions are elongated by ∼0.2 and ∼0.8%, respectively, confirming that Be_5_C_2_ monolayer indeed has negative Poisson's ratios.

## Discussion

The unusual negative Poisson's ratio may endow Be_5_C_2_ monolayer with enhanced toughness and shear resistance, as well as enhanced sound and vibration adsorption. Correspondingly, Be_5_C_2_ monolayer could find some important applications in the fields of mechanics, tissue engineering and national security. It is worth noting that the negative Poisson's ratio is rather peculiar, as in nature nearly all materials have a positive Poisson's ratio, except some so-called auxetic materials^59^. Recently, Jiang et *al.*^60^ demonstrated theoretically that single-layer black phosphorus has a negative Poisson's ratio due to the unique puckered configuration. However, the negative Poisson's ratio of phosphorene was observed in the out-of-plane direction, which is different from that of Be_5_C_2_ monolayer. Remarkably, the Poisson's ratio of Be_5_C_2_ monolayer in the *b* direction (−0.16) is much higher than that of phosphorene (−0.027)^60^, rendering Be_5_C_2_ monolayer a more promising candidate for specific application in mechanical devices. No doubt, the negative Poisson's ratio of Be_5_C_2_ monolayer should be originated from its intriguing structural properties, especially the uniquely oriented chemical bonds. Our results could provide some guidelines for designing materials with a negative Poisson 's ratio.

With so many fascinating properties, it is desirable to synthesize Be_5_C_2_ monolayer in the laboratory. Considering that there is no layered structure of Be_5_C_2_ in nature, a promising approach is to grow Be_5_C_2_ monolayer on the surface of metal or metal oxide via chemical vapour deposition with accurately controlled Be/C ratio, just similar to the growth of silicene^47^,^48^. It is noteworthy that Be has toxic properties and the chemical vapour deposition synthesis usually requires high temperature; hence, special caution should be given during the experimental realization.

To summarize, inspired by the bonding characters of a ppC-containing molecule, Be_9_C_2_^4−^, we designed a ppC-containing 2D inorganic material with a Be_5_C_2_ stoichiometry. Our DFT computations demonstrated that ppCs in Be_5_C_2_ monolayer are essentially stabilized by the charge transfer from Be ligands. The moderate cohesive energy, absence of imaginary modes in phonon spectrum and high melting point evaluated from FPMD simulations indicated that Be_5_C_2_ monolayer is experimentally viable. Especially, a global minimum search revealed that Be_5_C_2_ monolayer is the lowest-energy structure for the Be_5_C_2_ stoichiometry in 2D space, which endows Be_5_C_2_ monolayer great possibility to be realized experimentally. Our computations demonstrated that Be_5_C_2_ monolayer is semi-metallic with a zero band gap in the electronic band structure. More interestingly, Be_5_C_2_ monolayer has rather intriguing mechanical properties featured with a negative Poisson's ratio. Therefore, Be_5_C_2_ monolayer is expected to have wide applications in electronics and mechanics. We hope our theoretical studies will promote the experimental realization of this novel material and attract more attentions on investigating nanomaterials with novel chemical bonding.

## Methods

### DFT computations

For the Be_9_C_2_^4−^ molecule, geometry optimizations, frequency analyses and electronic structure computations were performed at the B3LYP^61^,^62^ level of theory with the 6–311+G*(*d*, *p*) basis set as implemented in Gaussian 03 package^63^. For 2D Be_5_C_2_ monolayer, DFT computations were performed using the plane-wave technique implemented in Vienna *ab initio* simulation package^64^. The ion–electron interaction was described using the projector-augmented plane wave approach^65^,^66^. The generalized gradient approximation expressed by PBE functional^67^ and a 500-eV cutoff for the plane-wave basis set were adopted in all the computations. The convergence threshold was set as 10^−4^ eV in energy and 10^−3^ eV Å^−1^ in force. We set the *x* and *y* directions parallel and the *z* direction perpendicular to the layer plane, and adopted a supercell length of 15 Å in the *z* direction. The Brillouin zones was sampled with an 8 × 6 × 1 *Γ* centred *k* points grid. The phonon spectrum was computed using finite displacement method as implemented in CASTEP code^68^. The elastic constants were also computed using the CASTEP code. The chemical bonding analysis of Be_5_C_2_ monolayer was done using the SSAdNDP method^46^, which can well interpret the chemical bonding in terms of classical lone pairs, two-centre bonds, as well as multi-centre delocalized bonds in bulk solids, surfaces and nanostructures.

### Molecular dynamics simulations

The thermal stability of Be_5_C_2_ monolayer was evaluated by means of first-principles molecular dynamics simulations. The ground-state structure of Be_2_C monolayer was annealed at different temperatures. At each temperature, MD simulation in NVT ensemble lasts for 10 ps with a time step of 1.0 fs. The temperature was controlled by using the Nosé-Hoover method^69^.

### Global minimum structure searches

The PSO method within the evolutionary scheme as implemented in the CALYPSO code^70^ was employed to find the low-energy structures of 2D Be_5_C_2_ monolayer. In our PSO simulation, the number of generation was maintained at 30. Unit cells containing total atoms of 7, 14 and 28 were considered. The structure relaxations during the PSO simulation were performed using Vienna *ab initio* simulation package at PBE level of theory.

### Data availability

All relevant data are available from the authors.

## Additional information

**How to cite this article:** Wang, Y. *et al*. Semi-metallic Be_5_C_2_ monolayer global minimum with quasi-planar pentacoordinate carbons and negative Poisson's ratio. *Nat. Commun.* 7:11488 doi: 10.1038/ncomms11488 (2016).

## Supplementary Material

Supplementary Information Supplementary Figures 1-5 and Supplementary Table 1
