# Supplementary material for: Semi-metallic Be5C2 monolayer global minimum with quasi-planar pentacoordinate carbons and negative Poisson's ratio
Source: Nat Commun. 2016 May 3;7:11488. doi: 10.1038/ncomms11488 (PMC4857406; doi:10.1038/ncomms11488)
Supplement: Supplementary Information — Supplementary Figures 1-5 and Supplementary Table 1 [file ncomms11488-s1.pdf]

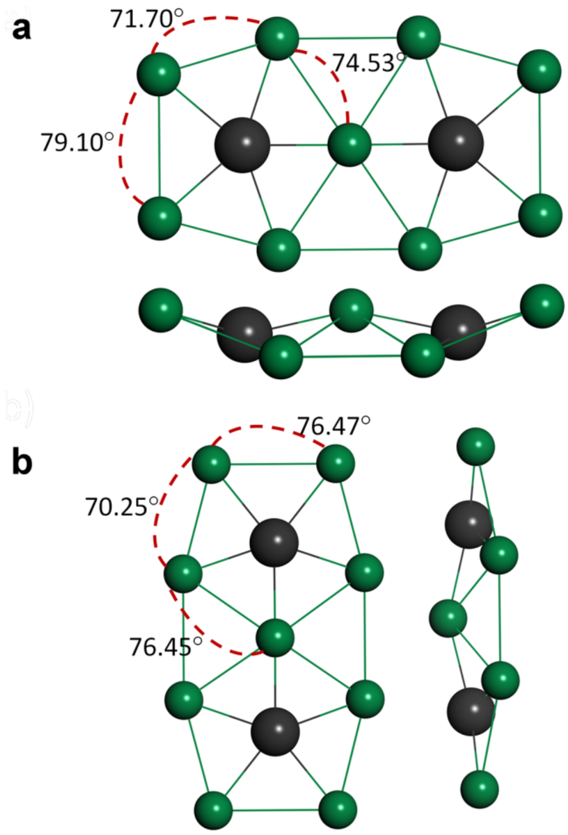

Supplementary Figure 1: Be_9C_2 moieties. Top and side views of Be_9C_2-I (a) and Be_9C_2-II (b) moieties in Be_5C_2 monolayer. The degrees of representative Be-C-Be angles are also given.

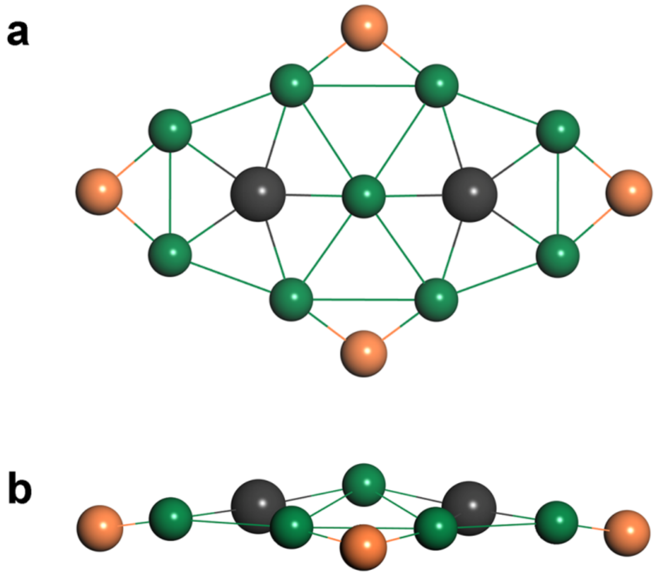

Supplementary Figure 2: $Be_9C_2H_4$ molecule. Top (a) and side (b) views of geometric structure of $Be_9C_2H_4$ molecule optimized at B3LYP/6-311+G*(d, p) level of theory. Yellow balls represent H atoms.

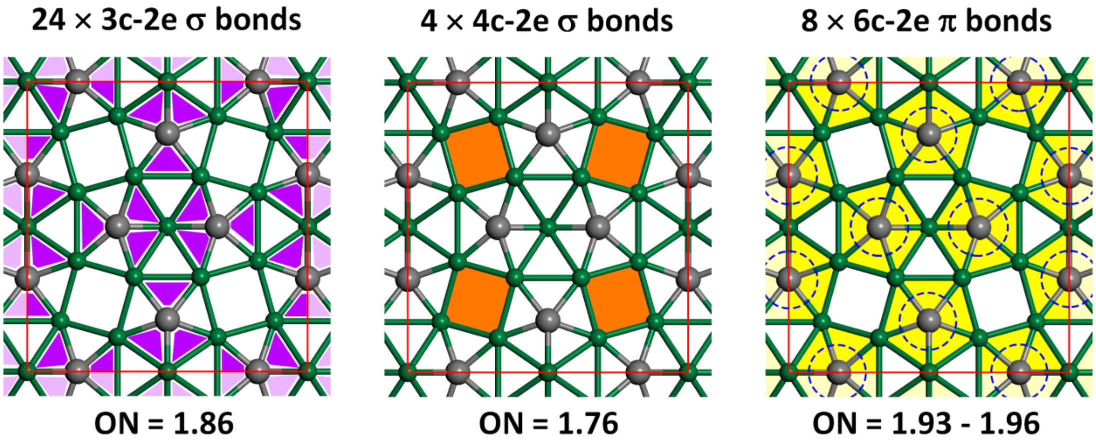

Supplementary Figure 3: Chemical bonding pattern. Schematic of SSAdNDP chemical bonding pattern for a unit cell of Be_5C_2 monolayer.

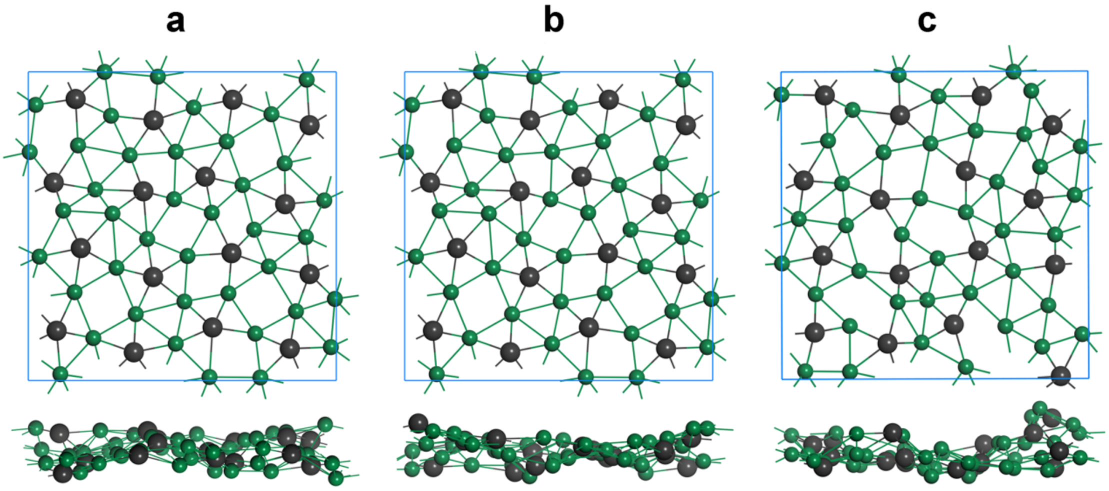

Supplementary Figure 4: Molecular dynamics simulations. A snapshot of the equilibrium structure of Be_5C_2 monolayer at (a) 1000 K, (b) 1500 K, and (c) 2000 K, respectively, at the end of 10 ps first-principles molecular dynamics simulation.

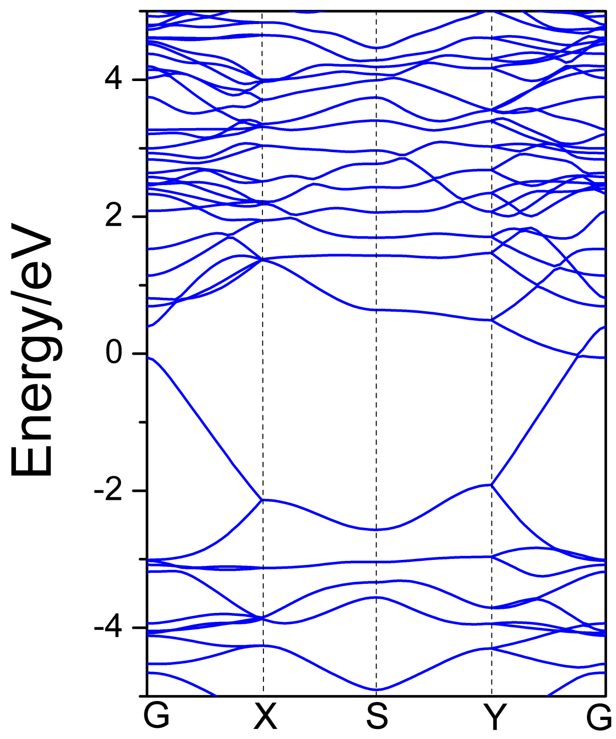

Supplementary Figure 5: Electronic properties of Be_5C_2 monolayer. Band structure of Be_5C_2 monolayer computed using HSE06 functional.

Supplementary Table 1: Optimized coordinates of the unit cell for Be_2C_5 monolayer.

Be_2C_5

1.0000000000000000			
8.9200000000000000	0.0000000000000000	0.0000000000000000	
0.0000000000000000	9.2060000000000000	0.0000000000000000	
0.0000000000000000	0.0000000000000000	15.0000000000000000	
Be	0.67587	0.88103	0.5114
Be	0.12065	0.17457	0.46288
Be	0.32413	0.11897	0.5114
Be	0.87935	0.82543	0.46288
Be	0.82413	0.38103	0.4886
Be	0.37935	0.67457	0.53712
Be	0.17587	0.61897	0.4886
Be	0.62065	0.32543	0.53712
Be	0.82413	0.61897	0.4886
Be	0.37935	0.32543	0.53712
Be	0.17587	0.38103	0.4886
Be	0.62065	0.67457	0.53712
Be	0.67587	0.11897	0.5114
Be	0.12065	0.82543	0.46288
Be	0.32413	0.88103	0.5114
Be	0.87935	0.17457	0.46288
Be	0	0	0.5091
Be	0.5	0.5	0.4909
Be	0.5	0	0.57173
Be	0	0.5	0.42827
C	0.5	0.82226	0.54597
C	0.5	0.17774	0.54597
C	0	0.32226	0.45403
C	0	0.67774	0.45403
C	0.68188	0.5	0.51428
C	0.31812	0.5	0.51428
C	0.81812	0	0.48572
C	0.18188	0	0.48572
